# User perceptions of avatar-based patient monitoring: a mixed qualitative and quantitative study

**DOI:** 10.1186/s12871-018-0650-1

**Published:** 2018-12-11

**Authors:** David W. Tscholl, Mona Weiss, Lucas Handschin, Donat R. Spahn, Christoph B. Nöthiger

**Affiliations:** 1 Institute of Anesthesiology, University Hospital Zurich, Raemistrasse 100, 8091 Zurich, Switzerland; 2 Leipzig University, Städtisches Kaufhaus, 04109 Leipzig, Germany

**Keywords:** Computer-assisted, Diagnosis, Patient monitoring, Situation awareness, Qualitative research

## Abstract

**Background:**

A new patient monitoring technology called Visual Patient, which transforms numerical and waveform data into a virtual model (an avatar) of the monitored patient, has been shown to improve the perception of vital signs compared to conventional patient monitoring. In order to gain a deeper understanding of the opinions of potential future users regarding the new technology, we have analyzed the answers of two large groups of anesthetists using two different study methods.

**Methods:**

First, we carried out a qualitative analysis guided by the “consolidated criteria for reporting qualitative research” checklist. For this analysis, we interviewed 128 anesthesiologists, asking: “Where do you see advantages in Visual Patient monitoring?” and afterward identified major and minor themes in their answers. In a second study, an online survey with 38 anesthesiologists at two different institutions, we added a quantitative part in which anesthesiologists rated statements based on the themes identified in the prior analysis on an ordinal rating scale.

**Results:**

We identified four high-level themes: “quick situation recognition,” “intuitiveness,” “unique design characteristics,” and “potential future uses,” and eight subthemes.

The quantitative questions raised for each major theme were: 1. “The Visual Patient technology enabled me to get a quick overview of the situation.” (63% of the participants agreed or very much agreed to this statement). 2. “I found the Visual Patient technology to be intuitive and easy to learn.” (82% agreed or very much agreed to this statement). 3. “The visual design features of the Visual Patient technology (e.g., the avatar representation) are not helpful for patient monitoring.” (11% agreed to this statement). 4. “I think the Visual Patient technology might be helpful for non-monitor experts (e.g., surgeons) in the healthcare system.” (53% of the participants agreed or strongly agreed).

**Conclusion:**

This mixed method study provides evidence that the included anesthesiologists considered the new avatar-based technology to be intuitive and easy to learn and that the technology enabled them to get an overview of the situation quickly. Only a few users considered the avatar presentation to be unhelpful for patient monitoring and about half think it might be useful for non-experts.

**Electronic supplementary material:**

The online version of this article (10.1186/s12871-018-0650-1) contains supplementary material, which is available to authorized users.

## Background

Patient safety is at risk if caregivers cannot perceive the patient’s vital signs, such as oxygen saturation or pulse rate. Unfortunately, the interface design of the current industrial standard patient monitoring does not optimally help the user to capture the essential information quickly [1–5].

In a previous study, we introduced a newly developed visualization technology called the Visual Patient (VP) that integrates the multitude of individual numeric and waveform monitoring data from conventional patient monitoring screens into a single visual monitor: a virtual animated model (or avatar) of the monitored patient. In the previous study, the VP technology enabled anesthesiologists to double the number of vital signs that they can perceive after a brief glance at the monitor versus conventional monitoring. At the same time, the anesthesiologists rated their confidence in the correctness of their diagnosis as higher and rated the perceived workload lower [3].

The purpose of developing the VP technology has been to enable care providers to understand the vast amount of vital sign information to improve their situational awareness [2, 4–6]. Endsley et al. describe situation awareness as “being aware of what is happening in a situation and understanding what that information means now and in the near future.” [1, 4, 7, 8] Loss of situation awareness makes good decision making impossible and plays a role in more than 75% of anesthesia and surgical adverse events [9–15].

With the development of new sensors, the manufacturers of patient monitors added more and more new indicators in a so-called single-sensor single indicator mode, thereby neglecting the human performance limitations of the users. The single-sensor single indicator mode means that data from individual sensors, such as the pulse frequency measured by the pulse oximetry sensor, are displayed one by one on a screen. This happens in the form of individual numbers or waveforms, which can also be arranged differently and have different colors depending on the device manufacturer. However, persons can only read numbers if they view them with foveal or sharp vision and foveal vision can only be directed at one number per time unit. After a person has read the first number on a monitor and mentally interpreted its meaning, which is further complicated by the similarity of the values of many numbers, the eyeballs can jump to a next number and read it. If the information is coded in colors and forms (as, e.g., in the patient avatar), several vital parameters more per time unit can be perceived simultaneously [2–4, 16].

With this study, we wanted to learn more about the opinions of anesthesia personnel (doctors and nurses) on the new avatar-based patient monitoring. The results of this study will be important to identify the strengths, weaknesses and capabilities of the technology.

## Methods

The Ethics Committee of the Canton of Zurich, Zurich, Switzerland, reviewed the study protocol and issued a declaration of no objection (BASEC No. Req-2016-00103). Nevertheless, all participants also gave their written consent to the anonymous use of their data. We conducted the qualitative part of this study according to the checklist “Consolidated criteria for reporting qualitative research.” [17]

### Study design

#### Study participants

For the initial, qualitative, part of this study, we interviewed predominantly anesthesia professionals from the anesthesia department of the University Hospital Zurich, Switzerland, a University hospital performing approximately 30.000 anesthesia procedures annually. One participant was from the anesthesia department of the Kantonsspital Winterthur, Switzerland, a teaching hospital performing approximately 10.000 anesthesia procedures per year.

For the second, quantitative part, we conducted an online survey and analyzed the ratings that participants from the same two centers gave on ordinal rating scales to statements that we derived from the first part of the study.

In both study steps, all participants were either staff or resident physicians, or nurse anesthetists. All staff physicians had an anesthesia board certification, and all nurse participants had completed their anesthesia specialization training. We recruited participants who responded to institutional e-mail invitations and additionally asked co-workers in person to participate according to their availability.

Most participants knew the data collectors personally before the study, as they worked in the same departments. We explained the purpose of the study, namely the evaluation of the novel avatar-based patient monitoring technology in the invitation e-mails and, when approaching a participant directly, in person.

### Part I: Qualitative analysis of interview answers

#### Study setup and data collectors

**Fig. 1:**
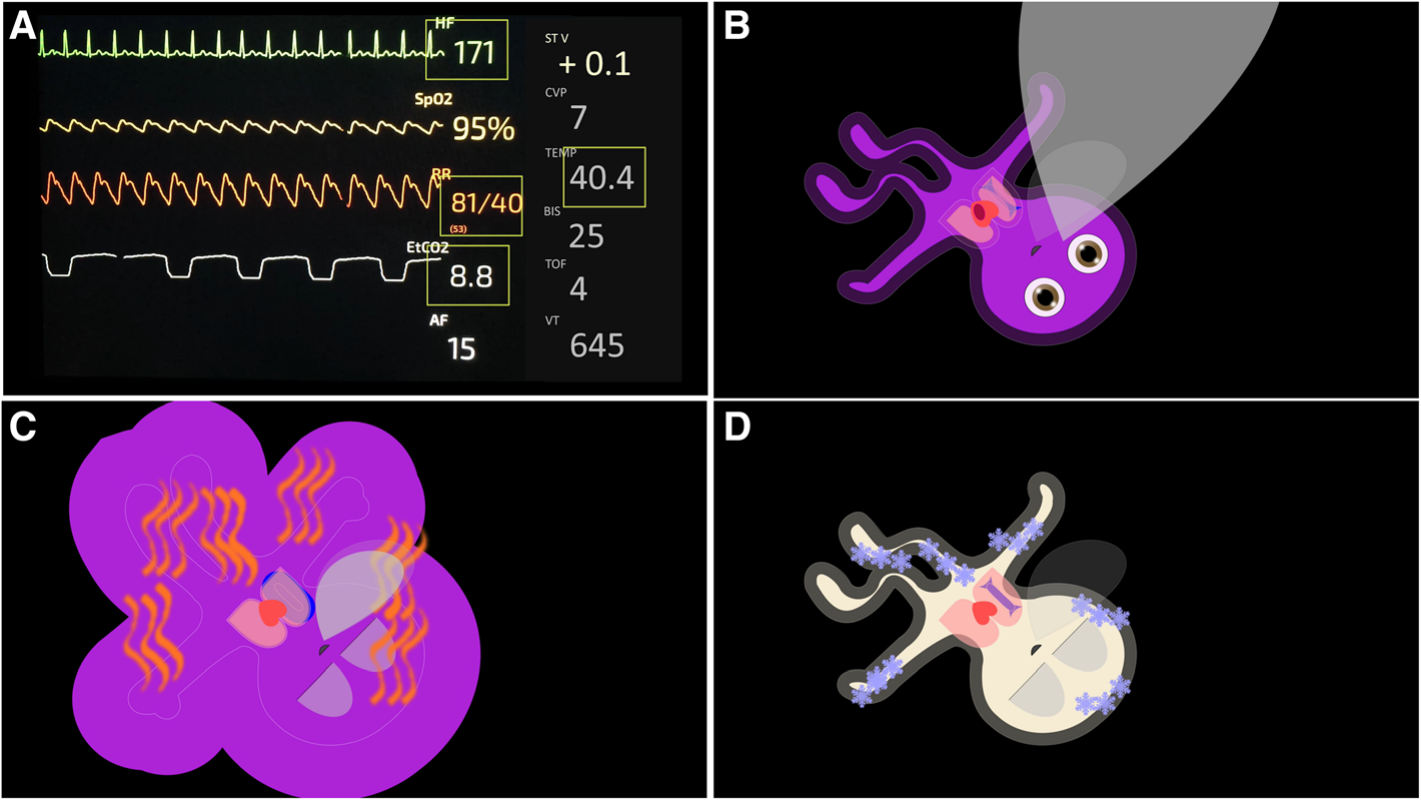
Graphical examples: **a** A state-of-the-art conventional patient monitoring interface with vital signs presented as individual numbers and wave-forms, i.e., a single sensor, single indicator philosophy. **b-d** Various patient states visualized using Visual Patient technology with vital signs displayed as direct visualizations, i.e., the visualizations represent what the information means in the form of an animated patient avatar, e.g., cyanotic skin color = low oxygen saturation. Pulse and respiratory frequency are not visible in a still picture, as their assessment requires an animation

We conducted the interviews for the first qualitative part of this study at the end of the data collection sessions that took place during the step-by-step development process of the VP technology. We also explained this step-by-step development process of VP technology in detail in a previous publication [3]. During the development process, the participants evaluated how they perceived the visualizations in iterative versions of the patient avatar. The evaluated versions differed only regarding the extensions and frequencies displayed in the avatar, but not in the design of the avatar itself. Besides, before each study session, participants completed a survey on personal data such as age, gender, anesthesia experience, etc., and watched an instructional video explaining the VP technology in detail. In total, each participant spent about half an hour per data collection and was able to gain experience with the VP during this time. Figure 1 shows a current, industry-standard patient monitoring interface and graphical examples of the VP technology.

Two data collectors, both medical doctors, conducted the interviews. One data collector (author LH) had completed his medical studies about 3 years before this study and was a 2nd-year anesthesia resident physician working 50% clinically and 50% scientifically at the University Hospital Zurich during the time of the study. He had previously completed an entry-level Good Clinical Practice (GCP) course offered by the clinical trials center at the University of Zurich and also participated in additional research projects during the time of the study.

The other data collector (author CBN) was a senior consultant doctor with > 20 years of anesthesia experience. He was working 100% clinically at the time of the study, had completed entry- and advanced-level GCP courses, and had previously participated in patient safety research projects.

#### Description of the interview

The interviews took place in different rooms of the University Hospital Zurich. During the interviews, we paid attention to an undisturbed environment, and no other persons than the data collector and the participant were present. The data collector initiated the semi-structured interviews by asking the question: “Which advantages do you see in the VP monitoring technology and why?” The participants were requested to answer the question candidly with whatever comes to their minds. The data collector recorded notes, typing along in a Microsoft Word (Microsoft Corp., Redmond, WA, USA) document on an Aspire V15 Nitro laptop computer (ACER, Inc., Taipei, Taiwan), while the participants verbalized their thoughts. There were no time constraints for answering, and the data collectors gave no prompts or guides. The transcript of the responses was visible to the participants during data entry and was made available at the end of the interviews for comments and corrections.

During the interview part, we asked a total of three questions. The present study analyses the participants’ responses to the first of these questions. The qualitative analysis of question number two: “What should we improve in the VP technology and how should these improvements look?” is analyzed in the supplementary material to the paper describing the comparative study [3]. We will separately report the analysis to question number three: “What are the most common problems with patient monitoring in your daily work?”

#### Analysis

To systematically analyze the interview responses, we first translated them from their original German language into English using Google Translate (Alphabet Inc., Mountain View, CA). Then we manually checked and corrected the output for meaning, syntax errors, and typographical errors and matched words with comparable meaning to facilitate word counting and coding. The words matched were: quick = rapid, fast, speedy; recognition = capture, acquire; assessment = analysis; situation = condition; vital parameter = vital sign. With the resulting adjusted English translation of the responses, excluding common English words such as the, and, e.g., etc., we conducted a word count and created a tag cloud using Wordle.net.

Following procedures for qualitative data analysis [17, 18] and using an exploratory thematic approach, we aimed to derive higher-level themes and subthemes from the responses. Two of the study authors (LH and DWT), both resident physicians, and one with previous experience in patient safety research conducted a two-stage process including deductive coding based on word count and inductive coding based on themes that emerged from the content of the interviews.

We outline and discuss these themes and sub-themes with examples in the results section, a table, and a coding tree.

Some individuals participated in two interviews because they attended more than one cycle of the step-wise development process of the technology and evaluated two versions of the avatar. In the analysis, we looked at their responses from both study sessions at once and consequently counted these participants only once, as if they had given just one interview.

We used Atlas TI 8.0 software (Scientific Software Development GmbH, Berlin, Germany) and Microsoft Word for data management.

### Part II: Quantitative analysis of statements rated in an online survey

#### Study setup

For the second, quantitative part of this study, we conducted an online survey of participants in two study centers who participated in a follow-up study on VP technology.

Like the participants in the first part of the study, these participants also received a structured introduction to the VP technology using a training video and then gained experience with the technology by evaluating different scenarios. We will report these studies separately.

After having taken part in the above-described study, participants received an e-mail invitation to anonymously participate in an online survey on the same day of their participation in the follow-up study. At the end of the follow-up study, we sent one reminder email to the participants to complete the online questionnaire.

#### Description of the online survey

In the online survey, we asked the participants a total of five questions, four of which we based on the topics identified in the qualitative analysis of the interview responses (part I of this study). Specifically, we created a statement for each of the main topics identified in the qualitative analysis of the interview responses. We considered the statements we created for the participants’ evaluation to be necessary for a better understanding of the technology and therefore wanted to examine them more precisely.

These statements were evaluated on five-point Likert scales by the new group of anesthesiologists from both centers. For the online survey, we used SurveyMonkey (SVMK Inc., San Matteo CA). The Likert scales had five divisions: “strongly disagree,” “disagree,” “neutral,” “agree,” and “strongly agree.”

#### Statistical analysis

We present the results of the online survey for each statement separately in the form of percentages as well as median and interquartile ranges (IQR). We used the Wilcoxon signed rank test to find out whether the sample medians were significantly different from neutral. We considered a difference from neutral as practically significant and a *p*-value of < 0.05 as statistically significant.

Through the evaluation of these statements, we wanted to quantify the agreement or disagreement of the participants with statements created from the interviews (part 1 of the study) by higher-level of evidence than purely qualitative description.

## Results

### Study and participant characteristics

A total of 158 anesthesia professionals took part in the iterative development process of the animated avatar. Of these participants, 11 did not give an interview, and 19 took part in two interviews, resulting in 128 individual interviewees.

Thirty-eight participants took part in the VP follow-up study, and 36 of them (95%) completed the online survey. Seven participants who participated in the interviews also participated in the follow-up study, resulting in a crossing-over between the interview participants and online survey participants of 21%.

All samples in both study steps were gender-, profession-, and experience balanced. Table 1 outlines the study and participant characteristics in detail.

**Table 1:** The study and participant characteristics in detail

	Part I: Iterative development process (participant Interview)	Part II: Visual Patient follow-up study (online survey)
Duration of study in days	248 (April 12th 2016 – December 16th 2016)	29 (September 20th 2018 – October 18th 2018)
Number of total participants	158	38
Number of participants who participated in both study parts	7	
Number of senior physicians (%)	49 (32%)	10 (26%)
Number of resident physicians (%)	57 (34%)	13 (34%)
Number of nurse anesthetists (%)	52 (34%)	15 (40%)
Number of female/male participants (%)	76 (48%) / 82 (52%)	21 (55%) / 17 (45%)
Number of participants according to study site USZ/KSW (%)	157 (99%) / 1 (1%)	16 (42%) / 22 (58%)
Median (IQR) age group of participants in years	35 to 44 (25 to 34–35 to 44)	35 to 44 (25 to 34–35 to 44)
Median (IQR) anesthesia experience group of participants in years	5 to 10 (1 to 5 – more than 10)	5 to 10 (1 to 5 – more than 10)
Number of individual participants who gave an interview or completed the online survey, respectively	128 (81%)	36 (95%)

*IQR* Interquartile range, *USZ* University Hospital of Zurich, *KSW* Kantonsspital Winterthur

### Part I: Qualitative analysis of interview answers

**Fig. 2:**
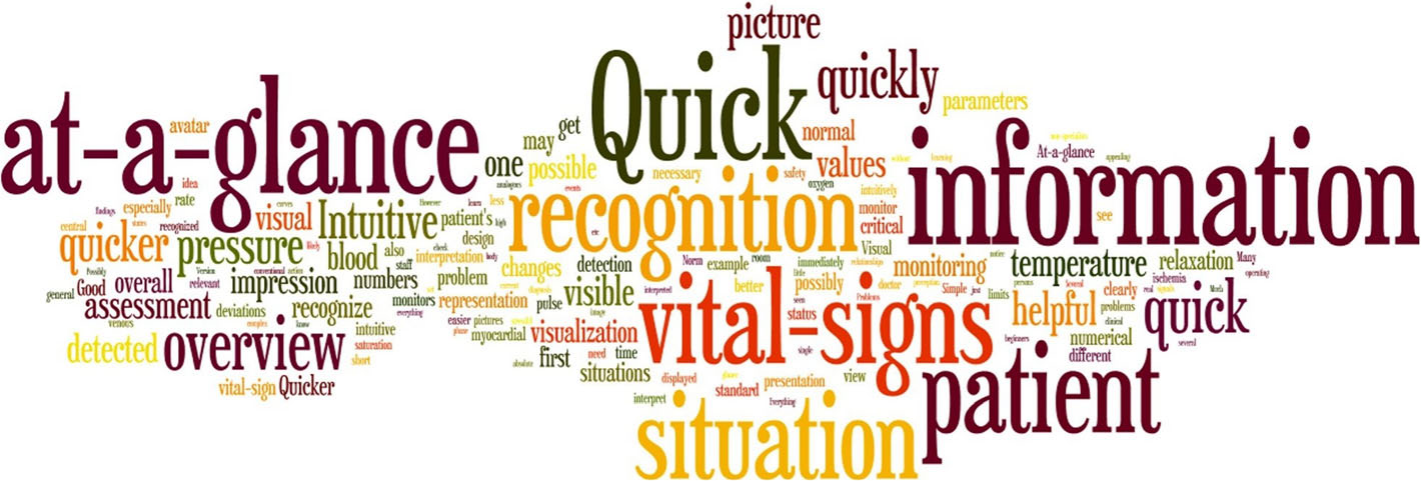
Tag cloud: A tag cloud (Wordle.net), created from the participants’ responses to quickly perceive the most prominent terms

The ten most frequently occurring words in the participants’ answers were: quickness/quick/quickly (72 participants, 56%), recognition/recognize (39 participants, 31%), at-a-glance (39 participants, 31%), information (35 participants, 28%), situation (33 participants, 26%), vital sign (30 participants, 23%), patient (28 participants, 22%), intuitive (21 participants, 16%), overview (19 participants, 15%), and picture (14 participants, 11%). Figure 2 provides the tag cloud created from the words the participants used in their responses.

From the word count, we identified the following high-level themes with subthemes: “quick recognition of situation” with the subthemes “at-a-glance information” and “visual diagnosis”; and “intuitiveness” with the subtheme “visual design.”

Additionally, we identified the following high-level themes with subthemes through inductive, free coding: “unique design characteristics” with the subthemes “single display,” “eye-catching,” “response stimulating,” and “absence of numbers”; and “potential future uses” with the subtheme “use by non-specialists.” The raw interview answers of the participants are provided in Additional file 1: Table S1 (raw interview answers). All themes and subthemes with participant counts, percentages and examples are outlined in Table 2 and shown graphically in Additional file 2: Figure S1 (coding tree).

**Table 2:** The high-level themes and subthemes with participant counts, percentages and examples

High-level theme	Subtheme	Examples
Quick recognition of situation (67 participants, 52%)		Participant #13: “Quick recognition of problems.”
		Participant #51: “Quick recognition of several vital signs simultaneously.”
		Participant #53: “Quick visualization of events and the (anatomic) location of the events.”
		Participant #111: “Quick recognition of relevant relationships, findings and situations.”
		Participant #112: “A picture, a glance and you have the overview.”
		Participant #132: “At a glance, holistic recognition of the situation.”
	At-a-glance information (42 participants, 33%)	Participant #14: “All parameters at a glance, simple presentation.”
		Participant #41: “It is easy to recognize vital signs as either “normal” or “abnormal.””
	Visual diagnosis (3 participants, 2%)	Participant #39: “Visual diagnosis is possible. Clearly arranged information.”
		Participant #62: “First impression of the patient in a moment.”
Intuitiveness (21 participants, 16%)		Participant #49: “Pretty intuitive. The design supports visual persons.”
		Participant #100: “Quick to learn. The instructional video is sufficient as an introduction.”
		Participant #101: “At a glance, intuitively, much more information than from a standard monitor can be gained.”
	Visual design (7 participants, 5%)	Participant #24: “You can interpret pictures quicker than numbers. Situations seem threatening or non-threatening at a glance.”
		Participant #59: “…no “translation” of numbers is needed.”
		Participant #89: “Color coding facilitates a quick assessment.”
		Participant #92: “…one does not need technical knowledge to understand the pictures.”
		Participant #99: “Possibly better visualization of vital signs through an avatar than through sober monitor curves.”
Unique design characteristics	Single display (12 participants, 9%)	Participant #16: “Most of the information previously separated into various numbers (sometimes distributed over several monitors) at a glance.”
		Participant #32: “Everything in one picture…”
	Eye-catching (6 participants, 4%)	Participant #8: “Problems are more eye-catching.”
		Participant #51: “Certain vital signs are very impressively displayed and immediately visible or better visible than in the conventional representation.”
		Participant #57: “Warning signals are more easily perceivable.”
		Participant #107: “Another way to attract the attention of the observer/user.”
	Response stimulating (5 participants, 4%)	Participant #6: “The display triggers an alarm reaction quickly.”
		Participant #95: “…especially with extreme deviations from the standard you have a strong internal need to take action.”
	Absence of numbers (5 participants, 4%)	Participant #5: “Intuitive recognition of the patient situation without becoming set on “numerical values.””
		Participant #86: “No number chaos.”
		Participant #89: “Less “scattered” data/numbers/values.”
Potential future uses (7 participants, 5%)		Participant #13: “…emergency situations.”
		Participant #66: “…especially in noisy surroundings.”
		Participant #70: “…trauma room…”
		Participant #90: “Safe in space, for airlines, cruises, on expeditions and in the military, a huge advantage.”
		Participant #124: “Basically, in stressful situations, one may be able to react more adequately to a visual image than to absolute (numerical) values that one must interpret first.”
		Participant #128: “A doctor, who monitors several operating rooms can immediately get an idea of a problem.”
	Use by non-specialists (7 participants, 5%)	Participant #8: “Simple interpretation also for interested non-specialists. (surgeons).”
		Participant #13: “Maybe helpful for beginners (with little monitoring experience).”
Further comments		Participant #10: “One glance from a distance enables the assessment of the patient situation.”
		Participant #54: “Rapid detection of the patient’s situation also in the ventilated and sedated patient (analogous to the clinical picture, as, for example, in the preclinical assessment).”
		Participant #79: “…different brain regions are activated in the users.”

*N* = 128

#### Themes

##### Quick recognition of situation

Of the 128 total participants, 110 participants (85%) made a comment that fit into either the theme “quick recognition of situation” or its subtheme “at-a-glance information.”

Participants commented that in the patient avatar much information is visible at a glance, which helps them to interpret the general patient situation more quickly, or as several participants put it, immediately get the “picture”. Several participants used the term “quick overview” in this context, and three participants reported that the VP enabled them to make a visual diagnosis. Others reasoned the technology helps to recognize changing patient states and become aware of problems more quickly because situations seem threatening or non-threatening at a glance. Three participants commented that relationships between vital signs might be perceived more quickly.

##### Intuitiveness

Twenty-one participants (16%) used the term “intuitive” in their responses. They used comments, such as: “pretty intuitive,” (Participant #42) or “intuitive presentation” (Participant #36). Several participants reported that the technology is easy to learn, and the instructional video was sufficient as an introduction. One participant reported that the visualizations do not need an explanation. Another participant said that with VP monitoring problems are perceived more implicitly: “you know that something is wrong before knowing exactly what and why” (Participant #76).

Seven of the participants indicated that they perceived the technology as intuitive because of its visual design. As possible explanations, in this context, participants pointed out that: “one does not need technical knowledge to understand the pictures” (Participant #92), and “no translation of numbers is needed” (Participant #59).

##### Unique design characteristics

Twenty-eight participants (22%) named unique design characteristics of the technology as advantages. We divided this theme into the four subthemes “single display,” “eye-catching,” “response stimulating,” and “absence of numbers.”

Several participants mentioned the fact that the VP technology includes all vital sign information into a single display, i.e., the patient avatar, which participants also called “picture” or “presentation.” Furthermore, participants reported that this renders the need to scan many numbers and waveforms on one or different monitors obsolete. “All information in a single place” (Participant #102).

Participants also pointed out that they perceived problems to be more eye-catching in an animated avatar because numbers and waveforms are not very catchy, and that the VP technology may serve to attract a care providers’ attention.

Some participants indicated that the design of the technology might cause them to act more quickly: “…you have a strong internal need to take action” (Participant #95), “…possibly quicker response to patient pathology” (Participant #76), and “The display triggers an alarm reaction quickly.” (Participant #6).

Furthermore, participants pointed out the absence of numbers repeatedly. “No number chaos.” (Participant #86), “Less scattered data/numbers/values” (Participant #89). One participant pointed out that the visual display may reduce the danger of becoming “set on numerical values” (Participant#5).

##### Potential future uses

The participants envisioned future uses of the technology would include situations where the cognitive load is high, for example, stressful and emergency situations, the trauma room, noisy surroundings, and places where a care provider monitors multiple patients. Participants also envisioned the VP to be used by non-anesthetist health care providers, with “surgeons” being named as an example (Participant #8), and by beginners less experienced in patient monitoring. Furthermore, the use of the technology for on-site training (Participant # 128), and in various places, for example, in space, on expeditions, etc. was suggested. In this context, participant #124 provided a possible explanation why the VP technology may have advantages under high cognitive workload: “One may be able to react more adequately to a visual image than to absolute (numerical) values that one must interpret first.”

##### Further comments

Some participants provided thoughts that did not fit into any of the existing categories. Participant #10 commented that VP monitoring could be used to monitor a patient from a distance. Participant #79 mentioned that the VP technology might engage different brain regions in the users during patient monitoring. Furthermore, the VP reminded participants of the clinical picture that they try to establish when evaluating a patient in the preclinical setting or at the first patient contact.

##### Critical comments

Five participants provided valuable critical comments. Two participants noted that the visual information, while quick to interpret, is not as precise as a number. The first impression of participant #58 was that the avatar looked “overloaded,” and another participant noted that the norm lines in the designs, while very helpful, at very high pulse and respiratory frequencies were sometimes difficult to detect.

One participant suggested that the technology should also feature trend monitoring.

### Part II: Quantitative analysis of statements rated in an online survey

The results of the assessments of the statements made according to the main topics identified in the qualitative analysis of the interviews (study part I) were as follows:

1. “The Visual Patient technology enabled me to get a quick overview of the situation.” Median response 3, IQR 2–3 (0 = strongly disagree, 1 = disagree, 2 = neutral, 3 = agree, 4 = strongly agree). Twenty-four of the 36 total participants (63%) agreed or strongly agreed to this statement.
2. “I found the Visual Patient technology to be intuitive and easy to learn.” Median response 3, IQR 3–4 (0 = strongly disagree, 1 = disagree, 2 = neutral, 3 = agree, 4 = strongly agree). Thirthy-one of 36 participants (82%) agreed or strongly agreed to this statement.
3. “The visual design features of the Visual Patient technology (e.g. the avatar representation) are not helpful for patient monitoring.” Median response 2, IQR 2–3 (0 = strongly disagree, 1 = disagree, 2 = neutral, 3 = agree, 4 = strongly agree). Four of 36 participants (11%) agreed to this statement
4. “I think the Visual Patient technology might be helpful for non-monitor experts (e.g. surgeons) in the healthcare system.” Median response 3, IQR 2–3 (0 = strongly disagree, 1 = disagree, 2 = neutral, 3 = agree, 4 = strongly agree). 19 of 36 participants (53%) agreed or strongly agreed to this statement.

**Fig. 3:**
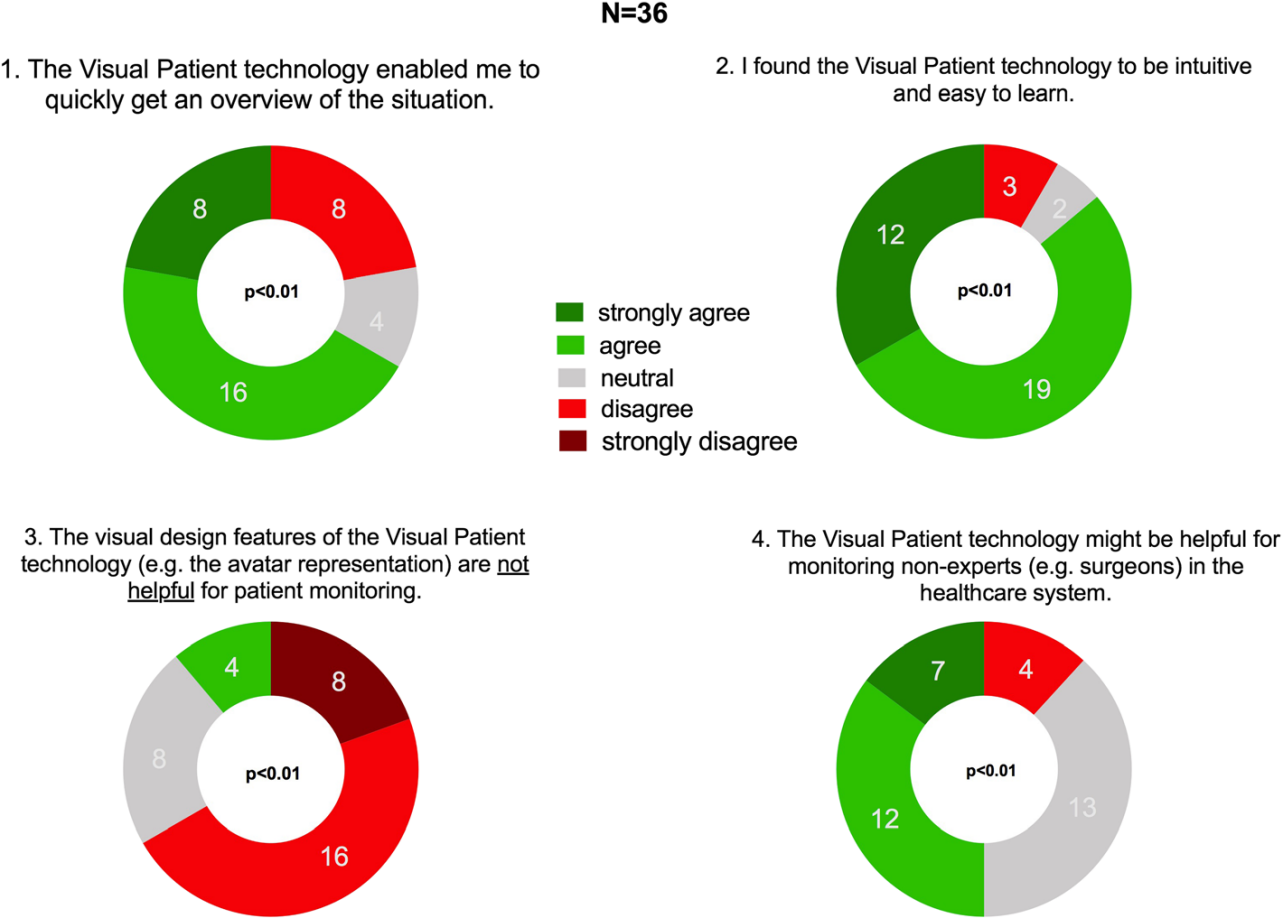
Presentation of the results of the quantitative online survey as donut charts with the number of participants who chose a particular category. We used the Wilcoxon signed rank test to find out whether the sample medians were significantly different from neutral

The sample medians of all four statements were statistically significantly different from neutral. Figure 3 shows donut diagrams of these results.

## Discussion

In this mixed method study, we first collected the opinions of a large group of anesthesiologists about a new avatar-based patient monitoring technology and divided them into topics using qualitative analysis. Based on these topics, in a second step, we prepared statements which we considered critical for a deeper understanding of the technology and had them quantitatively evaluated by a new large group of anesthesiologists.

In their responses to the first statement, two-thirds of the participants indicated that the new technology helps them get a quick overview of the situation. Endsley and Jones describe just this as the goal of a successful situation awareness system: “…a system interface concept that provides the operator with the necessary information as quickly as possible and without excessive cognitive effort” [1]. Improving situation awareness is crucial to enhance patient safety, as situation awareness errors are significant causes of perioperative morbidity and mortality [9–12] and patient monitoring information is an important source of situation awareness when caring for a patient. The users’ opinion in this respect is consistent with the results of the comparative study, where it was effectively proven that more vital signs could be perceived per time unit using the new technology [3].

Another important finding of this study is that users found VP technology to be intuitive and easy to learn. Intuitiveness is the characteristic that enables the use of technology using unconscious processing using stored experiential knowledge [19]. Cognitive ease in learning a new technology is crucial in introducing new technologies. An intuitive user interface creates confidence in the technology and is critical to user adoption. The results of the comparative study showed that VP technology does indeed have features associated with intuitiveness, as participants were able to achieve better results after watching the 6-min instructional video than with conventional monitoring, with which all participants had extensive experience [3].

Only 4 of 36 participants agreed with the statement that the visual design features (e.g., patient avatar) of the visual patient are not helpful for patient monitoring. The majority considered the visual design features of the new technology to be helpful. Research has highlighted the importance of cognitive absorption in the context of the introduction of new technologies. Cognitive absorption includes cognitive states such as focused immersion, increased pleasure, control, and curiosity and is an essential precursor of perceived usability and utility [20]. The fact that several participants described the design of the VP as an eye-catcher and emphasized its visual effects suggest that these design features may have led to a state of high cognitive absorption, which in turn may have improved performance and acceptance. In this respect, we would like to highlight some of the comments made by the subjects in the qualitative analysis. For example, the respondents mentioned: the lack of numbers, the integration of all parameters into a single image, the strong alarm response caused by the visual representation and the ability to make visual diagnoses. The ability to make visual diagnoses reminded some of the subjects of the treatment of real patients, where the first glance at a patient can already give the experienced clinician a lot of information.

Half of the participants who completed the online survey agreed with the statement that the new technology could be helpful for user groups who are not experts in patient monitoring. In the qualitative analysis, the participants imagined that the most significant benefit of the technology would be in stressful situations. It seems, in line with previous research, that a technology that lowers the cognitive burden required to gain situational awareness would indeed be most helpful in situations where the workload is high or cognitive capacity is low. Such situations have been shown to be prone to cause errors [1, 21–25].

In the interviews, the participants provided ideas for some exciting new hypotheses, e.g., whether avatar-based monitoring affects different brain areas and if so, how this relates to the results. Such a hypothesis could be tested using functional magnetic resonance imaging and could provide imaging data to correlate with the qualitative and quantitative results [26].

Whether a future avatar-based monitoring technology may be successfully introduced in the future OR or not depends crucially on the acceptance of the users and whether they consider a product useful. This study shows that the users attributed these properties to the VP technology. The key findings are that most users found it easy to learn to use the technology, had the impression that they quickly got a situation overview from it, and liked its visual design characteristics.

### Limitations

This study has limitations. In both study parts, the study participants were not randomly selected but consisted of samples of anesthesiologists who responded to institutional e-mail invitations and other participants that we recruited according to availability. However, the balanced samples of study centers, gender, occupation (doctors and nurses), and the high participation rate in the interviews and the online survey, respectively reduce the likelihood of selection bias.

In this study, we asked participants about the advantages they see in VP monitoring and not the disadvantages. Therefore, this analysis focused on the positive aspects that users see in the technology. We asked another question: “What should we improve in the Visual Patient?” within the same interviews to enable the participants to specifically target critical points. The evaluation of this question enabled numerous improvements of the technology in later versions of the avatar as described in detail in the comparative study [3]. Finally, we conducted the interviews and online surveys immediately after the first contact with VP technology. Therefore, the opinions reflect the benefits that users envisioned after their first contact with the technology. Future research should look at how perceptions would change after gaining real-life experience with the technology in a clinical setting.

## Conclusions

A large group of anesthesiologists identified benefits and possible future applications of the technology in semi-structured interviews. A second large group of anesthesiologists quantified agree- or disagreement with statements about VP technology derived from the interviews in an online survey. We discovered that the anesthesiologists considered avatar-based monitoring to be an intuitive technology that allowed them to get an overview of the patient’s condition quickly. The subjects found the visual design features (avatar representation) of the technology useful and about half of the subjects could imagine that the technology could be useful for non-experts. These are guiding insights for future development, research, and potential usage areas of the technology.

## Additional files

Additional file 1: **Table S1.** Raw interview answers: The raw data, translation and coding of the interview responses given by the participants to the question: “Which advantages do you see in the Visual Patient monitoring technology and why?”. (DOCX 293 kb)

Additional file 2: **Figure S1.** Coding tree: The coding tree with themes and subthemes identified from the interview transcripts with participant counts and percentages. We identified the themes underscored in red through deductive coding based on word-counts and the remaining through inductive, free coding. *N* = 128. (DOCX 177 kb)

## Abbreviations

GCP: Good clinical practice
IQR: Interquartile range
VP: Visual Patient
