# Supplementary material for: User perceptions of avatar-based patient monitoring: a mixed qualitative and quantitative study
Source: BMC Anesthesiol. 2018 Dec 11;18:188. doi: 10.1186/s12871-018-0650-1 (PMC6290504; doi:10.1186/s12871-018-0650-1)
Supplement: Supplementary file 1 — Table S1. Raw interview answers: The raw data, translation and coding of the interview responses given by the participants to the question: “Which advantages do you see in the Visual Patient monitoring technology and why?”. (DOCX 293 kb) [file 12871_2018_650_MOESM1_ESM.docx]

Additional file 1 for manuscript:

**User perceptions of avatar-based patient monitoring: a mixed qualitative and quantitative study.**

**Table S1:** The raw data, translation and coding of the interview responses given by the participants to the question: “Which advantages do you see in the Visual Patient monitoring technology and why?”

Rows two and three are presented in their original, unaltered form to enable traceability, and, therefore, contain typos and syntax errors.

In row four, “adjusted English translation”, words with comparable meaning were matched to facilitate word counting and coding: quick = rapid, fast, speedy; recognition = capture, acquire; assessment = analysis; situation = condition; vital parameter = vital sign.

| Visual Patient avatar version 1: | | | | |
| --- | --- | --- | --- | --- |
|  | Original, unaltered German answer | Unaltered Google Translate English translation | Adjusted English translation | Coding |
| 1 | Viele Info auf einen Blick. | Many info at a glance. | Much information at a glance. | - At a glance information |
| 2 | Schnelle Informationsübermittlung. Man bekommt rasch ein Bild. | Fast information transfer. You get a picture quickly. | Quick information transfer.  You get a picture quickly. | - Quick recognition of situation |
| 3 | Intuitivität. Schnelle Erfassung von vielen verschiedenen Zuständen. | Intuitiveness. Quick capture of many different states. | Intuitiveness. Quick recognition of many different patient states. | - Intuitiveness - Quick recognition of situation |
| 4 | Wichtigste Parameter in einem animierten Bild.  Hypertonus «springt ins Auge», initial von allem Anderen ablenkend, aber zwingt zum Überprüfen des Restes (ggfs unter Beizug «Zahlenmonitoring»)  Temperatur auffällige Darstellung  Relaxation gut ersichtlich -> wie messbar (TOFWatch?)  Version two: Schnelle Information über verschiedene Parameter auf einen Blick | Most important parameters in an animated picture. Hypertonic "jumps in the eye", initially distracting from everything else, but forces you to check the rest (if necessary with the help of "Number monitoring") Temperature conspicuous representation Relaxation clearly visible -> how measurable (TOFWatch?)  Version two: Quick information about various parameters at a glance | Most critical vital signs displayed in an animated picture.  Visualization for hypertension is striking, initially distracting from everything else, but forces you to check the rest (if necessary with the help of "numerical monitoring")  Temperature visualization is conspicuous.  Relaxation visualization is clearly visible -> how is it measured (TOF Watch?).  Version two: Quick information about various vital signs at a glance. | - At a glance information - Eye catching - Response stimulating |
| 5 | Schnelles Erfassen des Patienten und der wichtigsten Probleme möglich.  Intuitives Design – das einzige, das wirklich einer Erklärung bedarf ist der Relaxationszustand.  Version two: Intuitive Erfassung des Patienten ohne sich auf «Zahlenwerte» zu versteifen | Quick capture of the patient and the main problems possible. Intuitive design - the only thing that really needs an explanation is the state of relaxation.  Version two: Intuitive acquisition of the patient without stiffening to "numerical values" | Quick recognition of the patient situation and the main problems possible.  Intuitive design - the only thing that needs an explanation is the status of relaxation.  Version two: Intuitive recognition of the patient situation without becoming set on "numerical values." | - Quick recognition of situation - Intuitiveness - Absence of numbers |
| 6 | Schnelle Interpretation, schnelles vbisuelles Wahrnehmen. Löst schnell eine Alarmreaktion aus.  Version two: Intuitive signale auf die ein profi reagiert, und dann sofort ins detail überprüfen kann, oder falls nötig sofortmasnahmen einleitne kann | Fast interpretation, fast visual perception. Triggers an alarm reaction quickly. Version two: Intuitive signals to which a professional responds, and then immediately check in detail, or if necessary initiate immediate measures | Quick interpretation, quick visual perception. The display triggers an alarm reaction quickly.  Version two: Intuitive signals to which professionals respond, and then immediately check in detail, or if necessary initiate immediate measures. | - Quick recognition of situation - Response stimulating |
| 7 | Viele Informationen auf einem Bildschirm (BIS, ZVD)  Ischämie Herz klar ersichtlich | Lots of information on one screen (BIS, ZVD) Ischemia heart clearly visible | Lots of information on one screen (Bispectral index, central venous pressure)  Myocardial ischemia is clearly visible. | - At a glance information - Single display |
| 8 | Direkte Erfassung aller relevanten Parameter in einem Bild.  Einfache Interpretation auch für interessierte Laien (Chirurgen)  Probleme «stechen» schneller ins Auge | Direct acquisition of all relevant parameters in a picture. Simple interpretation also for interested laymen (surgeons) Problems "sting" faster. | Direct recognition of all relevant vital signs in a picture.  Simple interpretation also for interested non-specialists. (surgeons)  Problems are more eye-catching. | - Quick recognition of situation - Non-specialist use - Single display - Eye-catching |
| 10 | Schneller Gesamteindruck. (Im Alltag viele Fehlalarme/Übermonitorisierung). Ein Blick genügt aus der Distanz zur Einschätzung des Patientenzustandes. | Fast overall impression. (Many false alarms / overmonitoring in everyday life). One glance suffices from the distance to the assessment of the patient's condition. | Quick overall impression. (Many false alarms / overmonitoring in everyday life). One glance from a distance enables the assessment of the patient situation. | - Quick recognition of situation |
| 11 | Schneller Überblick. Intuitiv. | Quick overview. Intuitive. | Quick overview. Intuitive. | - Quick recognition of situation - Intuitiveness |
| 12 | Sehr prägnante Information. Schnelle Informationsaufnahme. | Very concise information. Fast information recording. | Very concise information. Quick recognition of information. | - At a glance information |
| 13 | Schnelle Erfassung von Problemem.  Version two: Schnelle beurteilung einer situation  v.a. in NF situationen  ev. Hilfreich für anfänger(mit wenig kenntnis, schnell veränderungen sehen. ) | Quick capture of problems.  Version two: Quick assessment of a situation V. A. in NF situations ev. Helpful for beginners (with little knowledge, see fast changes.) | Quick recognition of problems.  Version two: Quick assessment of a situation.  Especially in emergency situations.  Maybe helpful for beginners (with little monitoring experience) to recognize changes quickly). | - Quick recognition of situation - Potential future use - Non-specialist use |
| 14 | Alle Parameter auf einen Blick, einfache Darstellung.  Version two: Hilfreiche Normwerte. Wobei: Was bedeutet Normalwert, gibt es nicht einen Norm-range? i.e. Blutdruck normal zwischen 90-120mmHg systolisch zB. | All parameters at a glance, simple presentation.  Version two: Helpful standard values. What is normal value, is not there a standard range? i.e. Blood pressure normal between 90-120mmHg systolic eg. | All parameters at a glance, simple presentation.  Version two: Indicators of normal values are helpful.  However, how are normal values defined, is not there a standard range? For example, blood pressure normal between 90-120mmHg systolic etc. | - At a glance information |
| 15 | Schneller kurzer Überblick über Gesamtsituation | Quick overview of the overall situation | A quick overview of the overall situation. | - Quick recognition of situation |
| 16 | Viel schnellerer Überblick.  Sehr interessante und zukunftsträchtige Idee.  Die meisten vorher in diverse Zahlen (z.T. auf mehreren Monitoren verteilt) aufgetrennte Informationen auf einen Blick. Auch zahlen oder Kurven, die sonst nicht sehr eingängig sind oder die ich sonst eher vernachlässige (ZVD, EKG) | Much faster overview. Very interesting and promising idea. Most of the information previously separated into various numbers (sometimes distributed over several monitors) at a glance. Also pay or curves that are otherwise not very catchy or that I tend to neglect otherwise (ZVD, ECG) | Much quicker overview.  Exciting and promising idea. Most of the information previously separated into various numbers (sometimes distributed over several monitors) at a glance. Also visualizes numbers or curves that are otherwise not very catchy or that I tend to neglect otherwise (central venous pressure, ECG). | - Quick recognition of situation - Single display - Eye-catching |
| 17 | mehrere Funktionen werden in einer Darstellung integriert | several functions are integrated in one presentation | Several vital signs are displayed in one presentation. | - At a glance information - Single display |
| 18 | Alle wichtigen Parameter auf einem Blick, visualisiert, somit einfacher aufzunehmen. Möglicherweise ist es eine einfache Variante, sich einen raschen, aber eher oberflächlichen Überblick über den Patienten zu verschaffen. | All important parameters at a glance, visualized, thus easier to record. It may be a simple option to get a quick, but rather superficial, view of the patient. | All critical vital signs are visualized at a glance and, therefore, easier to recognize. It may be a simple option to get a quick, but rather superficial, view of the patient. | - Quick recognition of situation |
| 19 | Intuitive Erfassung des Zustandes/Problemes (auch für Laien). | Intuitive recording of the condition / problem (also for laymen). | Intuitive recognition of the patient situation / problem (also for non-specialists). | - Intuitiveness - Quick recognition of situation - Non-specialist use |
| 20 | Trendverläufe visualisieren  Version two: Eher hilfreich, wenn ein «Trend» angezeigt wird, als ein «ist» Zustand von wenig komplexen Werten (Herzfrequenz, ZVD).  Bei komplexeren Zusammenhängen ggf. hilfreich (Zusammenspiel HZV, MAP, Widerstand, Füllung) | Visualize trends  Version two: Helpful when a "trend" is displayed as an "is" state of less complex values (heart rate, ZVD). For more complex contexts, if necessary, helpful (interplay of CO, MAP, resistance, filling) | Visualize trends.  Version two: Instead of a "is" status, a "trend" would be more helpful for less complex values, e.g., heart rate, central venous pressure.  Also, possibly helpful for more complex contexts (interplay of cardiac output, mean arterial pressure, resistance, volume status). |  |
| 21 | Nach Eingewöhnung mit einem Blick verschiedene Daten erfassbar. | After familiarization with a glance, various data can be detected. | After familiarisation, various data can be perceived at a glance. | - At a glance information |
| 21 | Schnelles Erkennen des allgemeinen Patientenzustandes | Quick recognition of general patient condition | Quick recognition of general patient situation. | - Quick recognition of situation |
| 23 | Viele Parameter auf einen Blick | Many parameters at a glance | Many vital signs are visible at a glance. | - At a glance information |
| 24 | Bilder kann man schneller beurteilen als Zahlen. Situationen wirken auf einen Blick bedrohlich oder nicht bedrohlich. | You can judge pictures faster than numbers. Situations seem threatening or non-threatening at a glance. | You can interpret pictures quicker than numbers. Situations seem threatening or non-threatening at a glance. | - Quick recognition of situation - Visual design |
| 25 | Schnelle/Intuitive Interpretation der einzelnen Parameter. | Fast / intuitive interpretation of the individual parameters. | Quick/intuitive interpretation of the individual parameters. | - Quick recognition of situation - Intuitiveness |
| 26 | Schnelleres Erkennen von Vital Parameter Veränderungen (auf einem Display) | Faster detection of vital parameter changes (on a display) | Quicker detection of vital sign changes (in a single display). | - At a glance information - Single display |
| 27 | Sehr intuitive/schnelle Erfassung der Informationen auf einen Blick. | Very intuitive / fast information capture at a glance. | Very intuitive/quick information recognition at a glance. | - At a glance information - Intuitiveness |
| 28 | Guter Überblick/Gesamteindruck über den Zustand eines Patienten. «Ludische» Komponente als sehr positiv empfunden/Ansprechend. | Good overview / overall impression of the condition of a patient. "Ludische" component as very positive / appealing. | Good overview/overall impression of the situation of a patient. Gamification component very positive/appealing. | - Quick recognition of situation |
| 29 | Intuitives Patientenmonitoring  Rascher Überblick | Intuitive patient monitoring Quick overview | Intuitive patient monitoring. Quick overview. | - Quick recognition of situation - Intuitiveness |
| 30 | Einfache, schnell interprierbare Information. Erfassung der wichtigsten VP in kurzer Zeit möglich. | Simple, quickly interpreting information. Capture the most important VP in a short time possible. | Simple, quick to interpret information. Recognize the most critical vital signs in a short time. | - At a glance information - Intuitiveness |
| 32 | Alles in einem Bild, schnelle Erfassung.  Version 2: Schnelles Erfassen relevanter Zusammenhänge und Befunde und Zustände | Everything in one picture, fast acquisition. Version 2: Quickly capture relevant relationships and findings and conditions | Everything in one picture, quick information recognition.  Version 2: Quickly recognize relevant relationships, findings and situations. | - Quick recognition of situation - Single display |
| 33 | Klar ersichtliche Parameter (Relaxation und Sauerstoffsättigung). | Clearly visible parameters (relaxation and oxygen saturation). | The visualizations of the vital signs relaxation and oxygen saturation. | - At a glance information |
| 34 | Leichteres erkennen von pathologischen Vitalparametern.  Version two: Schnelles Bemerken von Pathologien. | Easier detection of pathological vital signs. Version two: Quick notice of pathologies. | Easier detection of pathological vital signs.  Version two: Quicker notice of pathologies. | - Quick recognition of situation |
| 35 | Schneller Überblick | Quick overview | Quick overview. | - Quick recognition of situation |
| 36 | zB ST-Strecken Deviation war gut sichtbar | eg ST-distance deviation was well visible | The visualization for myocardial ischaemia is clearly visible. | - At a glance information |
| 37 | Intuitive Darstellung | Intuitive presentation | Intuitive presentation | - Intuitiveness |
| 38 | keine absoluten Werte | no absolute values | No absolute (numerical) values. | - Absence of numbers |
| 39 | «Blickdiagnose» möglich. Übersichtlicher. | "Eye diagnosis" possible. Clearer. | Visual diagnosis is possible. Clearly arranged information. | - Visual diagnosis |
| 40 | Alle Informationen auf einen Blick | All information at a glance | All information at a glance. | - At a glance information |
| 41 | dichotome Parameter sind einfacher zu erkennen | dichotomous parameters are easier to recognize | It is easy to recognize vital signs as either “normal” or “abnormal”. | - At a glance information |
| 42 | Einfacher Überblick. Ziemlich intuitiv. Unterstützt die visuell «interpretierenden» Personen.  Version two: Situation wird auf einen Blick erfasst. Intuitiv. | Simple overview. Pretty intuitive. Supports the visually "interpretive" persons.  Version two: Situation is captured at a glance. Intuitive. | Simple overview. Pretty intuitive. The design supports visual persons.  Version two: Situation is recognized at a glance. Intuitive. | - Quick recognition of situation - Intuitiveness |
| 43 | Kann mir gut vorstellen, dass eine Visualisierung der Vitalparameter, wie sie in diesem Projekt geplant ist, vom Gehirn schneller erfasst werden kann als die Vielzahl abstrakter Zahlen, welche wir normalerweise auf unseren Monitoren zu sehen bekommen, insbesondere, wenn es – z. B. für einen Kaderarzt, der frisch in den Raum kommt – darum geht, sich möglichst schnell einen Ueberblick zu verschaffen. | I can well imagine that a visualization of the vital signs, as planned in this project, can be detected more quickly by the brain than the multitude of abstract numbers that we normally see on our monitors, especially if it is - for example. For example, for a cadre doctor who comes fresh into the room - it's about getting as quickly as possible an overview. | I can imagine that a visualization of the vital signs, as planned in this project, can be detected more quickly by the brain than the multitude of abstract numbers that we usually see on our monitors. For example, for a doctor who comes into an operating room for the first time it is essential to get an overview as quickly as possible. | - Quick recognition of situation - Potential future use |
| 44 | Alle Parameter auf einen Blick sichtbar  Version two: Einfach gestaltete Dinge wie Temperatur, Relaxation sind sehr eindeutig, gut und schnell zu erkennen | All parameters visible at a glance  Version two: Simply designed things like temperature, relaxation are very clear, good and quick to recognize | All vital signs are visible at a glance.  Version two: Simply designed things like temperature and relaxation may be precisely and quickly recognized. | - Quick recognition of situation |
| 45 | Alle Informationen auf einen Blick. | All information at a glance. | All information at a glance. | - At a glance information |
| 46 | Schneller Zugang zur Information bei vital gefährdeten Patienten. | Quick access to information in vitally endangered patients. | Quick access to information in vitally endangered patients. | - At a glance information |
| 47 | Intuitiv, Schnell Erlernbar, Gute Übersicht über die Situation | Intuitive, Quick to learn, Good overview of the situation | Intuitive, quick to learn. Good overview of the situation. | - Quick recognition of situation - Intuitiveness |
| 48 | Auf einen Blick alle Parameter ersichtlich. | At a glance all parameters are visible. | At a glance, all vital signs are visible. | - At a glance information |
| 49 | Schnelles Erfassen von grossen Veränderungen der Vitalparameter. | Quick capture of major changes in vital signs. | Quick recognition of major vital sign changes. | - Quick recognition of situation |
| 50 | auf 1 Blick sehen, ob alles i.o. oder nicht | at 1 glance, see if everything i.o. or not | At a glance, you see if everything is okay or not. | - Quick recognition of situation |
| 51 | -schnelleres Erfassen der wichtigen Abweichungen von Vitalparameter  - schnelles Erfassen mehrerer Vitalparameter gleichzeitig  - Vereinfachung für Anfänger, die Gesamtlage zu erkennen  Version two: gewisse Parameter sind sehr eindrücklich dargestellt und sofort sichtbar (Temperatur) bzw. besser sichtbar wie bei der konventionellen Darstellung | -faster detection of important deviations from vital signs - fast acquisition of several vital signs simultaneously - Simplification for beginners to recognize the overall situation  Version two: certain parameters are very impressively displayed and immediately visible (temperature) or better visible as in the conventional representation | -quicker detection of important vital sign deviations.  - quick recognition of several vital signs simultaneously.  - Simplification for beginners to recognize the overall situation.  Version two: certain vital signs are very impressively displayed and immediately visible (e.g., temperature) or better visible than in the conventional representation. | - Quick recognition of situation - Non-specialist use - Single display - Eye-catching |
| 52 | schnelleres Bemerken von Veränderungen der Vitalparameter | faster notice of changes in vital signs | Quicker notice of vital sign changes. | - At a glance information |
| 53 | schnelle Visualierung vom Geschehen und Ort vom Geschehen.  Wenn man aus der Materie kommt man einen schnelleren Blick auf das Geschehen, muss aber die Abbildungen kennen. | fast visualization of events and location from the events. When you get out of matter you get a quicker view of the action, but need to know the pictures. | Quick visualization of events and the (anatomic) location of the events.  You get a quicker view of the action, but you need to know the pictures. | - Quick recognition of situation |
| 54 | schnelles Erfassen des Patientenzustands (analog zum klinischen Bild zB in der präklinischen Beurteilung) auch beim beatmeten und sedierten Patienten | rapid detection of the patient's condition (analogous to the clinical picture eg in the preclinical assessment) also in the ventilated and sedated patient | Rapid detection of the patient's situation also in the ventilated and sedated patient (analogous to the clinical picture, as, for example, in the preclinical assessment). | - Quick recognition of situation |
| 55 | Eyecatcher. Alle Informationen auf einen Blick. | Eyecatcher. All information at a glance | Eye-catching. All the information at a glance. | - At a glance information - Eye-catching |
| 56 | Schnelligkeit; auf einen Blick ein «Bauchgefühl» bekommen. | Speed; get a "gut feeling" at a glance. | Quickness; get a "gut feeling" at a glance. | - Quick recognition of situation |
| 57 | - Bild auf einen Blick - Warnsignale werden einfacher wahrgenommen | - Picture at a glance - Warning signals are perceived easier | - The picture at a glance.  - Warning signals are more easily perceivable. | - Quick recognition of situation - Eye-catching |
| 58 | Idee eines Avatars grundsätzlich begrüssenswert. In der Realität: erster Eindruck = «zu überladen!». | Idea of an avatar basically welcome. In reality: first impression = «overload!». | The idea of an avatar is welcome. However, in reality: first impression = «overload!». |  |
| 59 | alles auf einen Blick, keine «Übersetzung» der Zahlen nötig | all at a glance, no «translation» of the numbers needed | All information at a glance, no «translation» of numbers is needed. | - At a glance information - Visual design |
| 60 | Viele Informationen, bei denen die genauen numerischen Werte eine geringe Rolle spielen, werden vereinfacht und auf einen Blick dargestellt. | Many pieces of information, in which precise numerical values play a minor role, are simplified and presented at a glance. | Many pieces of information, in which precise numerical values play a minor role are simplified and presented at a glance. | - At a glance information |
| 61 | Schnelle Info auf einen Blick (bei Atmung oder Frequenzenextreme schwierig)  Version two: Es erhöht meiner Meinung nach die Sicherheit , da es nicht nur Zahlen sind sondern visuell das Problem auch erkennbar ist. | Quick info at a glance (difficult when breathing or frequency extremes)  Version two: In my opinion, it increases security, as it is not just numbers but visually the problem is also noticeable. | Quick information at a glance (challenging to recognise during breathing or frequency extremes).  Version two: In my opinion, it increases safety, as it is not just numbers, but the problem is also noticeable in a visual form. | - Quick recognition of situation |
| 62 | Erster Eindruck über den Patienten in «einem Augenblick»  Körpertemperatur, ST Strecke, | First impression of the patient in «one moment» Body temperature, ST stretch, | First impression of the patient in a moment.  Body temperature, myocardial ischemia indicator. | - Quick recognition of situation - Visual diagnosis |
| 63 | Schnelle Uebersicht; wenig zusätzliche Info | Quick overview; little additional info | Quick overview; little additional information. | - Quick recognition of situation |
| 64 | Nach kurzer Anlernphase, leichtere/schnellere Erfassung des Patientenzustandes  Version two: Normbegrenzungen hilfreich. | After a short learning phase, easier / faster recording of the patient's condition  Version two: Norm limits helpful. | After a short learning phase, easier / quicker recognition of the patient's situation.  Version two: Norm limits helpful. | - Quick recognition of situation |
| 65 | Gute Idee. Schnelle Erfassung auf einen Blick. | Good idea. Fast acquisition at a glance. | Good idea. Quick information recognition at a glance. | - At a glance information |
| 66 | Schnelle Beurteilung möglich, vorallem auch in lauter Umgebung  Version two: Besseres Monitoring durch Darstellung der Normal/Grenzbereiche | Quick assessment possible, especially in noisy surroundings  Version two: Better monitoring by displaying the normal / border areas | A quick assessment is possible, especially in noisy surroundings.  Version two: Better monitoring by displaying the normal / border areas. | - Quick recognition of situation - Potential future use |
| 67 | Schneller Überblick über Patientenzustand. Visuelle Darstellung der integrierten Parameter | Quick overview of patient condition. Visual representation of the integrated parameters | A quick overview of patient situation. The visual representation of integrated vital signs. | - Quick recognition of situation |
| 68 | Evtl einzelne Partien des VP als «Alarmunterstreichung» einblenden, analog zum aktuellen Blinken des pathologischen Wertes bei Alarmierung auf den Dräger monitoren | Possibly show individual parts of the VP as "alarm underline", analogous to the current flashing of the pathological value when alerting to the Dräger monitors | Improvement suggestion: Show individual elements of the avatar as "alarm underline", analogous to the current flashing of the pathological value in Dräger monitors. |  |
| 69 | Einige Vitalparameter sind sehr gut sehr schnell ersichtlich (Temperatur, Relaxation, Blutdruck). | Some vital signs can be seen very quickly (temperature, relaxation, blood pressure). | Some vital signs can be recognized very quickly (temperature, relaxation, blood pressure). | - At a glance information |
| 70 | Gibt Überblick in stressigen Situationen, wo noch keine Ordnung herrscht (z.B. Schockraum -> fokusiert auf Patient, kann Respi nicht sehen usw.)  Simpel | Provides overview in stressful situations where there is still no order (for example, shock room -> focused on patient, can not see Respi, etc.) Simple | Provides an overview in stressful situations when there is no situation awareness yet, for example, trauma room -> focus is on the patient and cannot see respirator, etc. | - Quick recognition of situation - Potential future use |
| 71 | Alle Infos auf einen Blick ersichtlich  Version two: guter Überblick | All information can be seen at a glance Version two: good overview | All information can be seen at a glance. Version two: good overview. | - Quick recognition of situation |
| 72 | Schnelle Übersicht über den Patientenzustand. | Quick overview of the patient's condition. | Quick overview of the patient's situation. | - Quick recognition of situation |
| 73 | Probleme können schnell erkannt werden. | Problems can be detected quickly. | Problems can be detected quickly. | - Quick recognition of situation |
| 74 | Schnelle Erfassung der Informationen | Fast acquisition of information | Quick recognition of information. | - At a glance information |
| 75 | Für Laien ggf. einfacher zu erfassen. | For laymen possibly easier to capture. | Possibly easier to recognize for non-specialists. | - Non-specialist use |
| 76 | Erkennen von möglichen Problemen auf einen Blick und eher intuitiv/implizit – man weiss dass etwas nicht stimmt ohne schon ganz genau zu wissen was und warum. Dadurch ggf. schnellere Reaktion auf Patientenpathologie und besseres Outcome für Patient?  Version two: Schnellere und intuitivere Wahrnehmung der allgemeinen Patientensituation | Recognizing possible problems at a glance and more intuitive / implicit - you know that something is wrong without knowing exactly what and why. As a result, possibly faster response to patient pathology and better outcome for patient?  Version two: Faster and more intuitive perception of the general patient situation | Recognizing possible problems at a glance and more intuitively/implicitly - you know that something is wrong before knowing exactly what and why. As a result, possibly quicker response to patient pathology and a better outcome for the patient?  Version two: Quick and more intuitive perception of the general patient situation. | - Quick recognition of situation - Intuitiveness - Response stimulating |
| 77 | Visuell ansprechend. | Visually appealing. | Visually appealing. |  |
| 78 | Alles in einem Bild, schnelle Erfassung. | Everything in one picture, fast acquisition. | Everything in one picture, quick information recognition. | - At a glance information - Single display |
| 79 | Geschwindigkeit der Erfassung (erster Eindruck), multimodale Informationen (Blick auf verschiedene Geräte entfällt), andere Hirnregionenen werden beansprucht ;-) | Speed of acquisition (first impression), multimodal information (view of different devices is not necessary), other brain regions are claimed ;-) | The quickness of information recognition (first impression), multimodal information (view of different devices is not necessary), different brain regions are engaged in the users ;-) | - At a glance information - Single display |
| 80 | Nach einer kurzen Trainingsphase ist die schnelle Erfassung des Patientenzustandes sehr gut möglich.  Version two: Design 1b mit den Normwerten als Referenz sind hilfreich  Blitzartige Erfassung der Gesamtsituation | After a short training phase, the quick recording of the patient's condition is very possible.  Version two: Design 1b with the standard values as reference are helpful Lightning capture of the overall situation | After a short training phase, the quick recognition of the patient's situation is possible.  Version two: In design version two the standard values as a reference are helpful.  Lightning-speed quick recognition of the overall situation. | - Quick recognition of situation |
| 81 | Visuelle interpretation der parameter gibt schnellen Gesamtüberblick über den Zustand des Patienten. | Visual interpretation of the parameters gives a quick overall view of the condition of the patient. | Visual interpretation of the vital signs gives a quick overall view of the situation of the patient. | - Quick recognition of situation |
| 82 | Gewisse Informationen sind sehr schnell « auf einen Blick « ersichtlich. (zB Blutdruck, Wachheit, Relaxation). | Certain information can be seen very quickly "at a glance". (eg blood pressure, alertness, relaxation). | Specific information can be seen very quickly "at a glance," e.g. blood pressure, alertness, relaxation. | - At a glance information |
| 83 | Statische Parameter sind sehr schnell erfassbar (i.e. Augen, Temperatur, ST-Deviation) | Static parameters can be detected very quickly (i.e. eyes, temperature, ST-deviation) | Static parameters can be detected very quickly, e.g., eyes, temperature, myocardial ischaemia. | - At a glance information |
| 84 | Dramatische Veränderungen des Zustandes des Patienten werden schnell erfasst.  Visuelle Darstellung kann evtl die Awareness eines kritischen Zustandes erhöhten (Bsp nicht messbarer RR kann als Artefakt oder aber real interpretiert werden, dies wird möglicherweise eher als real eingestuft bei hypotonem visual patient) | Dramatic changes in the condition of the patient are quickly detected. Visual presentation may increase the awareness of a critical condition (eg un measurable RR may be interpreted as an artifact or real, this may be more likely to be considered real in hypotonic visual patient) | Dramatic changes in the situation of the patient are quickly detected.  Visual presentation may increase the awareness of a critical situation, e.g. unmeasurable blood pressure may be interpreted as an artefact or real, this may be more likely considered real in a hypotonic avatar. | - Quick recognition of situation - Response stimulating |
| 85 | Man kann mehr Faktoren auf einen Blick erfassen. | You can capture more factors at a glance. | You can recognize more vital signs at a glance. | - At a glance information |
| 86 | Schnelleres Erfassen/Erblicken von gewissen Abweichungen  kein Zahlen – Chaos | Faster detection / seeing of certain deviations no numbers - mess | Quicker detection/seeing of specific deviations.  No number chaos. | - At a glance information - Absence of numbers |
| 89 | - Schnelle visuelle Erfassung der Vitalfunktionen - Vitalparameter werden in einem Bild zusammengefasst - Farbliche Kodierungen erleichtern eine schnelle Einschätzung - Weniger «zerstreute» Daten/Zahlen/Werte | - Fast visual capture of vital signs - Vital parameters are summarized in a picture - Color codings facilitate a quick assessment - Less "scattered" data / numbers / values | Quick visual recognition of vital signs:  - Vitals are summarized in a picture.  - Color codings facilitate a quick assessment.  - Less "scattered" data/numbers/values. | - At a glance information - Absence of numbers - Visual design |
| 90 | - Für ungeübte Personen ergibt sich ein verständliches Bild des Patienten (vorausgesetzt die ungeübte Person hat das Minitoring-System korrekt auf den Patienten eingestellt). Sicher in Raumfahrt, für Fluglinien, Schiffsreisen, bei Expeditionen und im Militär ein sehr grosser Vorteil. - Wenn man nicht direkt den Patienten überwachen muss, sondern z.B. als OA mehrere Monitorbilder im Kontrollraum (Büro) verfolgt oder auf einer IPS, sieht man intuitiv wo ein Problem ist. | - For inexperienced persons, a clear picture of the patient results (provided that the untrained person has set the minitoring system correctly on the patient). Safe in space, for airlines, cruises, on expeditions and in the military a very big advantage. If one does not have to monitor the patient directly, but e.g. when OA is tracking multiple monitor images in the control room (office) or on an IPS, you can intuitively see where a problem is. | - For inexperienced persons, a clear picture of the patient results (provided that the untrained person has set the monitoring system correctly on the patient). Safe in space, for airlines, cruises, on expeditions and in the military, a huge advantage.  If one does not monitor the patient directly, but, for example, when a doctor is tracking multiple monitors in a control room (office) or on an intensive care unit, they can intuitively see where a problem is. | - Non-specialist use - Intuitiveness - Potential future use - Quick recognition of situation |
| 91 | Intuitiv, schnell erfassbar | Intuitive, quickly detectable | Intuitive, quickly detectable information. | - Intuitiveness - At a glance information |
| 92 | Vorteile liegen klar im Verständniss, man braucht kein grosses fachliches Wissen um die Bilder zu verstehen.  Nachteil wenn man nie auf den Monitor schaut dann schaut man so eher auch nicht. | Advantages are clear in the understanding, one does not need a large technical knowledge to understand the pictures. Disadvantage if you never look at the monitor then you do not look that much. | Advantages manifest in understanding: one does not need technical knowledge to understand the pictures.  Disadvantage: if you never look at the monitor, then this changes nothing. | - Visual design |
| 93 | Alle Informationen auf einen Blick | All information at a glance | All information at a glance. | - At a glance information |
| 94 | Schnelle Informationsgewinnung in kürzerer Zeit (als mit konventionellem Monitoring)  Version two: Normwerte sind hilfreich, nicht zu unübersichtlich | Fast information retrieval in less time (than with conventional monitoring)  Version two: Norm values are helpful, not too confusing | Quick information retrieval in less time (than with conventional monitoring).  Version two: Norm values are helpful, not too confusing. | - At a glance information |
| 95 | Sehr intuitiv, prima vista schnelles Einschätzen von Puls und Blutdruck, gerade bei starken Abweichungen von den Normparametern hat man ein starkes internes Handlungsbedürfnis | Very intuitive, great speed and blood pressure assessment, especially with strong deviations from the standard parameters you have a strong need for internal action | Very intuitive, at a glance assessment of pulse and blood pressure assessment, especially with extreme deviations from the standard you get a strong internal need to take action. | - Intuitiveness - Quick recognition of situation - Response stimulating |
| 96 | Möglicherweise besseres Visualisieren durch das Männchen als durch die nüchternen Monitorkurven | Possibly better visualization by the male than by the sober monitor curves | Possibly better visualization of vital signs through an avatar than through sober monitor curves. | - Visual design |
| 98 | Gute Übersicht über den Zustand des Patienten  Wärmezustand des Patienten sehr intuitiv erfassbar  Insgesamt ansprechendes Symbolbild „Mensch“ | Good overview of the condition of the patient Warming condition of the patient can be detected very intuitively Overall appealing symbol image "human" | Good overview of the situation of the patient.  A high body temperature of the patient can be detected very intuitively.  Overall appealing symbol image of a "human." | - Quick recognition of situation - Intuitiveness |
| 99 | «Klinifizierung» des Monitorings. Blickdiagnostik. | «Clinification» of monitoring. View diagnostics. | «Clinification» of patient monitoring. Visual diagnosis. | - Visual diagnosis |
| 100 | Schnell erlernbar. Instructional video genügt als Einführung. | Quick to learn. Instructional video is sufficient as an introduction. | Quick to learn. The instructional video is sufficient as an introduction. | - Intuitiveness |
| 101 | Schnelle Erfassung vom aktuellen Zustand des Patienten  Nicht nur Linien, Zahlen und Kurven, sondern bildliche Darstellung  Version two: Auf einen Blick intuitiv viel mehr Info als auf normalem Monitor. | Quick acquisition of the current condition of the patient Not just lines, numbers and curves, but pictorial representation  Version two: At a glance, intuitively much more info than on a normal monitor. | The quick recognition of the current situation of the patient.  Not just lines, numbers and curves, but a pictorial representation.  Version two: At a glance, intuitively, much more information than from a standard monitor can be gained. | - Quick recognition of situation |
| 102 | alle Infos an einem Ort | all information in one place | All information in a single place. | - Single display |
| 103 | Schnelles Erkennen von Abweichungen. Übersicht auf einen Blick. | Quick recognition of deviations. Overview at a glance. | Quick recognition of deviations. Overview at a glance. | - Quick recognition of situation |
| 104 | intuitive schnellere Aufnahme der VP und Schwere der Erkrankung | intuitive faster recording of VP and severity of the disease | Intuitive, quicker recognition of vital signs and severity of the disease. | - Intuitiveness - Quick recognition of situation |
| 105 | rasch erster Eindruck | quick first impression | Quick first impression |  |
| 106 | Für wichtigste Parameter wie RR, HF und SpO2 sehr intuitiv  Körpertemperatur auch | For most important parameters like RR, HF and SpO2 very intuitive Body temperature too | Very intuitive for most critical vital signs, like blood pressure, pulse rate, oxygen saturation and body temperature. | - Intuitiveness |
| 107 | Weitere Möglichkeit die Aufmerksamkeit des Beobachter/Ueberwacher auf sich zu ziehen. | Another way to attract the attention of the observer / monitor. | Another way to attract the attention of the observer/user. | - Eye-catching |
| 108 | Extremzustände schnell ersichtlich. Wärmezustand sehr einprägsam. Wachheit idem.  Version two: Statische Parameter (zB Temperatur, Wachheitsgrad, Relaxation) lassen sich whs schneller erfassen. Bei den dynamischen Parametern (zB Blutdruck) nicht ganz so klar, uU Lernzeit notwendig | Extremely fast states. Heat state very memorable. Alertness idem.  Version two: Static parameters (eg temperature, wakefulness, relaxation) can be detected faster whs. With the dynamic parameters (eg blood pressure) not quite as clear, possibly learning time necessary | Extremely quick states (pulse rate, respiratory rate) are quickly recognizable. Temperature status is very memorable. Alertness idem.  Version two: static vital signs, e.g. temperature, wakefulness, relaxation, can likely be detected quicker. The dynamic vital signs, e.g., blood pressure, are not quite as clear, possibly learning time is necessary. | - Quick recognition of situation |
| Visual Patient avatar version 2: | | | | |
| 109 | rasche Entscheidung kritisch / unkritisch durch visuelle Darstellung von normal vs nicht-normal die im Endeffekt eine zügigere „serielle“ Analyse erlaubt | rapid decision critical / uncritical by visual representation of normal vs non-normal which in the end allows a more speedy "serial" analysis | Quick decision critical/uncritical through visual representation of normal and non-normal vital signs, which in the end allows for a quicker assessment. | - Quick recognition of situation - Visual design |
| 110 | Es können mehrere Parameter auf einen Blick erfasst werden – eventuell kann so die Sicherheit gesteigert werden – bzw. das Gesamtbild des Patienten besser und schneller erfasst werden. | Several parameters can be recorded at a glance - possibly increasing safety - or the overall picture of the patient can be recorded better and faster. | Several parameters can be perceived at a glance - possibly increasing safety - or the overall picture of the patient can be understood better and quicker. | - Quick recognition of situation |
| 111 | Schnelles Erfassen relevanter Zusammenhänge und Befunde und Zustände | Quick capture of relevant relationships and findings and conditions | Quick recognition of relevant relationships, findings and situations. | - Quick recognition of situation |
| 112 | Ein Bild, ein Blick und man hat die Übersicht. | A picture, a look and you have the overview. | A picture, a glance and you have the overview. | - Quick recognition of situation - Single display |
| 113 | Schneller Überblick | Quick overview | Quick overview. | - Quick recognition of situation |
| 114 | Alle Parameter auf einem Blick | All parameters at a glance | All vital signs at a glance. | - At a glance information |
| 115 | Referenzen vorhanden. Parameter klar ersichtlich. | References available. Parameter clearly visible. | References available. Vital sings clearly visible. |  |
| 116 | Situation wird auf einen Blick erfasst. Intuitiv. | Situation is detected at a glance. Intuitive. | The situation is detected at a glance. Intuitive. | - Quick recognition of situation - Intuitiveness |
| 117 | auf einen Blick sehr viele Informationen, gleich klinischer Eindruck beim ersten Patientenkontakt | At a glance, a lot of information, the same clinical impression at the first patient contact | A lot of information at a glance. Similar to the clinical impression at the first patient contact. | - At a glance information |
| 119 | schnelles Erfassen er Vitalparameter, visueller Eindruck ob in Normbereich bzw. Normbereiche gleich mitgezeigt.  Zum neuen Design: Normwertgrenzen sind sehr hilfreich. Bei sehr hohem Puls/AF zum Teil schwierig zu Erkennen -> evt bei Peak BD/Puls die Grenze aufleuchten lassen? | fast acquisition of vital signs, visual impression whether shown in standard range or standard ranges.  About the new design: Norm value limits are very helpful. At very high pulse / AF sometimes difficult to detect -> evt at peak BD / pulse let the limit light up? | Quick recognition of vital sign information, visual impression whether a vital is within or outside of its normal range.  About the new design (Version 2): The norm value limits are very helpful but at very high pulse/respiratory frequencies sometimes difficult to detect.  Possible solution: emphasize the norm line at peak pulse / blood pressure. | - At a glance information |
| 120 | Gute Visualisierung und evtl. Schnellere Erfassung der Gesamtsituation | Good visualization and possibly faster capture of the overall situation | Good visualization and possibly quicker recognition of the overall situation. | - Quick recognition of situation |
| 121 | Ein Monitoring für die wichtigsten Informationen zum Allgemeinzustand des Patienten. | A monitoring for the most important information about the general condition of the patient. | A monitor showing the most essential information about the general situation of the patient. | - Quick recognition of situation |
| 122 | schnellere Erfassung des Patientenzustands, vor allem durch weniger erfahrenes Personal | Faster detection of the patient's condition, especially by less experienced staff | Quicker detection of the patient's situation, especially by less experienced staff. | - Quick recognition of situation - Non-specialist use |
| 123 | Alle Informationen auf einen Blick, resp. kritische Situationen können schnell, jedoch unspezifisch erfasst werden | All information at a glance, resp. Critical situations can be detected quickly, but nonspecifically | All information at a glance.  Critical situations can be detected quicker, but nonspecifically. | - Quick recognition of situation |
| 124 | Grundsätzlich kann man in Stresssituationen evtl. adäquater auf ein visuelles Bild reagieren als auf absolute Werte, die man interpretieren muss. Schnellere Beurteilung möglich auf einen Blick. | Basically, in stressful situations, one may be able to react more adequately to a visual image than to absolute values that one has to interpret. Faster assessment possible at a glance. | Basically, in stressful situations, one may be able to react more adequately to a visual image than to absolute (numerical) values that one must interpret first. Quicker assessment possible at a glance. | - Quick recognition of situation - Potential future use |
| 125 | Normbegrenzungen hilfreich. | Norm limits helpful. | Norm limits are helpful. |  |
| 126 | No response |  |  |  |
| 127 | Alle Informationen auf einen Blick | All information at a glance | All information at a glance | - At a glance information |
| 128 | Patienen Sicherheit, indem der OA, der ein paar Säle überwacht sich sofort ein Bild vom Problem machen kann.  Teaching und Problembesprechung mit jungen Ärzten. | Patients safety by the OA, who monitors a few rooms immediately can get an idea of the problem. Teaching and problem discussion with young doctors. | Patient safety: A doctor, who monitors several operating rooms can immediately get an idea of a problem. Teaching and problem discussion with young doctors. | - Potential future use - Quick recognition of situation |
| 129 | No response |  |  |  |
| 130 | Rascher Überblick, vor allem Kombination von Farben undBewegungen in einer Figur sehr hilfreich. | Quick overview, especially combination of colors and movements in a figure very helpful. | Quick overview, especially the combination of colors and movements in the avatar is helpful. | - Quick recognition of situation |
| 131 | No response |  |  |  |
| 132 | Visuelle Darstellung der Hämodynamik und Vitalwerte, keine Zahlen | Visual presentation of hemodynamics and vital signs, no numbers | The visual representation of hemodynamics and vital signs, no numbers. | - Absence of numbers |
| 133 | Auf Einen Blick Situation gesamtheitlich erkennen | At a glance, recognize the situation holistically | At a glance, holistic recognition of the situation. | - Quick recognition of situation |
| 134 | Einige Parameter wie ZVD, Ischämie, Pulsoxymetrie und Temperatur können rasch erfasst und interpretiert werden. | Some parameters such as ZVD, ischemia, pulse oximetry and temperature can be quickly detected and interpreted. | Some parameters such as central venous pressure, myocardial ischemia, oxygen saturation and temperature can be quickly detected and interpreted. | - At a glance information |
| 135 | No response |  |  |  |
| 136 | Schnelle Information über verschiedene Parameter auf einen Blick | Quick information about different parameters at a glance | Quick information about different vital signs at a glance. | - At a glance information |
| 137 | No response |  |  |  |
| 138 | guter Überblick | good overview | good overview | - Quick recognition of situation |
| 139 | Intuitive Patienteneinschätzung, Vitalparameter sind visuell schnell einschätzbar und auf einen Blick im Fokus, ohne über den gesamten Monitor schweifen zu müssen. | Intuitive patient assessment, vital signs can be assessed quickly and visually at a glance, without having to worry about the entire monitor. | Intuitive patient assessment, vital signs can be assessed quickly and visually at a glance, without having to scan the entire monitor. | - Intuitiveness - At a glance information - Visual design - Single display |
