# Supplementary material for: User perceptions of avatar-based patient monitoring: a mixed qualitative and quantitative study
Source: BMC Anesthesiol. 2018 Dec 11;18:188. doi: 10.1186/s12871-018-0650-1 (PMC6290504; doi:10.1186/s12871-018-0650-1)

**Figure S1: Coding tree:** The coding tree with themes and subthemes identified from the interview transcripts with participant counts and percentages. We identified the themes underscored in red through deductive coding based on word-counts and the remaining through inductive, free coding. N=128


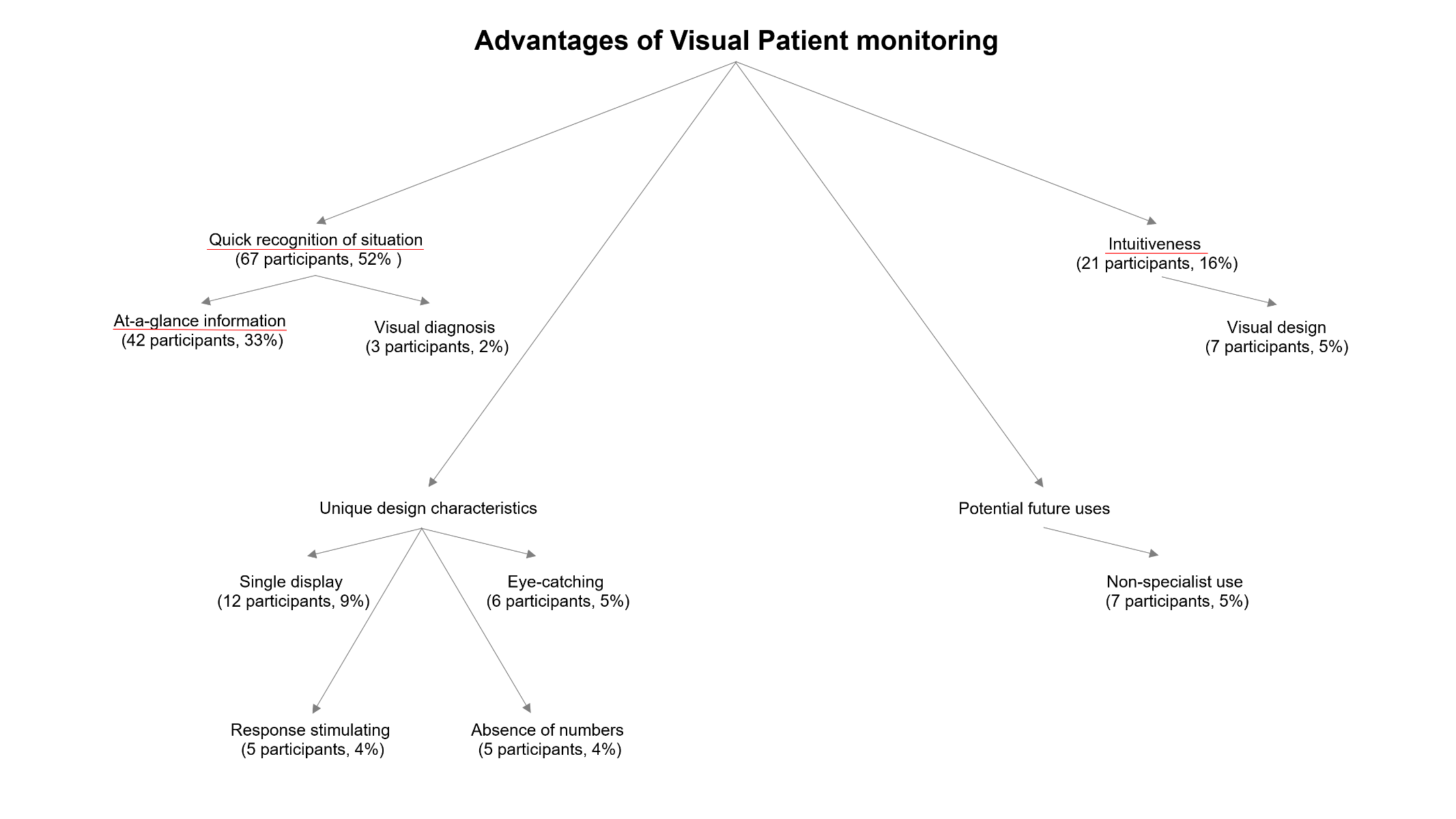

Supplement: Supplementary file 2 — Figure S1. Coding tree: The coding tree with themes and subthemes identified from the interview transcripts with participant counts and percentages. We identified the themes underscored in red through deductive coding based on word-counts and the remaining through inductive, free coding. N = 128. (DOCX 177 kb) [file 12871_2018_650_MOESM2_ESM.docx]
